# How did states in the United States adapt their cancer control plan in response to the COVID‐19 pandemic?

**DOI:** 10.1002/puh2.179

**Published:** 2024-05-29

**Authors:** Jason Semprini

**Affiliations:** ^1^ Department of Epidemiology University of Iowa College of Public Health Iowa City Iowa USA

**Keywords:** cancer, COVID‐19, equity, policy, population health, prevention, screening, state, treatment

## Abstract

The COVID‐19 pandemic upended the delivery of cancer services across the care continuum. By outlining specific strategies for addressing cancer in the state, cancer control plans serve a critical role during a public health emergency. This policy analysis aims to understand how states updated their cancer control plan as a response to COVID‐19. All plans from 50 states and the District of Columbia were reviewed for language related to “COVID.” Among the 51 cancer plans analyzed, 7 plans met the inclusion criteria (Illinois [IL], Iowa [IA], Maine [ME], Nevada [NV], North Carolina [NC], Utah [UT], and Vermont [VT]). These seven plans adapted their cancer control plan in response to the COVID‐19 pandemic across three main themes: (1) improving care across the cancer care continuum, from prevention to screening and treatment; (2) improving cancer care service delivery by expanding telehealth, addressing workforce shortages, and investing in public health systems; and (3) achieving population health equity by addressing social determinants of health. Two states only adapted their plans by prioritizing future monitoring and evaluation activities as related to the COVID‐19 pandemic (ME and VT). The other five states all took different approaches to improve cancer care by adapting their service delivery and addressing social determinants of health. IL prioritized access to cancer screenings through expanding equity informed telehealth models. IA also prioritized equitable screenings as well as clinical trial participation, by addressing workforce shortages. NV focused on prevention, leveraging telehealth and specifically targeted food security and job loss resulting from the pandemic. NC‐directed cancer treatment efforts by addressing workforce shortages. UT integrated telehealth and equity initiatives to combat barriers like food insecurity and social disparities. Continued policy surveillance is needed to ensure that patients receive timely, appropriate cancer care during future public health emergencies. Research evaluating whether these plan adaptations improved outcomes or advanced equity remains warranted.

## INTRODUCTION

The COVID‐19 pandemic upended the delivery of cancer services across the care continuum [1, 2, 3, 4, 5]. The pandemic also led to health policy changes at federal, state, and local levels [6]. At the intersection of these levels of government are state comprehensive cancer control plans [7]. State comprehensive cancer control plans are funded by the federal government and developed by state health departments in collaboration with healthcare providers, public health agencies, and community organizations. By outlining specific goals and strategies for addressing cancer in the state, these plans serve a critical function during a public health emergency.

The goal of the State Cancer Control Plan program is to help states reduce the burden of cancer by promoting evidence‐based interventions and addressing disparities in cancer care. The CDC provides technical assistance and funding to support the development and implementation of state cancer control plans. By working together, states can develop comprehensive approaches to cancer prevention, early detection, and treatment that can improve the health of their communities. The plans outline specific goals and strategies for addressing cancer in the state and may include initiatives such as increasing access to cancer screening, promoting healthy behaviors, and supporting cancer research and treatment.

There are no specific requirements for state cancer control plans, as each state has the flexibility to develop a plan that meets the needs of its community [7, 8]. States have used cancer control plans to implement a wide range of innovative policies and programs. For example, some states have used their plans to increase access to cancer screening, promote healthy behaviors, and support cancer research and treatment [9, 10, 11]. Other states have focused on addressing disparities in cancer care and improving the quality of cancer care for underserved populations [12].

Including COVID‐19 in a state cancer control plan could be beneficial for several reasons. First, the COVID‐19 pandemic has had a significant impact on the healthcare system, and addressing it in the plan can help ensure that cancer prevention, early detection, and treatment efforts are not disrupted. Second, the pandemic has highlighted the importance of addressing disparities in healthcare and ensuring that underserved populations have access to quality care. Including COVID‐19 in the plan can help states focus on these issues and develop strategies to address them.

Additionally, addressing COVID‐19 in the cancer control plan can help states coordinate their response to the pandemic with their broader efforts to address cancer. For example, a state may want to prioritize screening and treatment for cancer patients who are at higher risk for complications from COVID‐19, or it may want to incorporate COVID‐19 prevention measures into its cancer prevention campaigns. By addressing COVID‐19 in the plan, states can ensure that their efforts to combat cancer are integrated with their response to the pandemic.

As we move beyond the emergency phase of the pandemic, this policy analysis aims to understand how states updated their cancer control plan as a response to COVID‐19.

## MATERIALS AND METHODS

To conduct a comprehensive qualitative policy analysis, an extensive review of cancer control plans from all 50 states in the United States, as well as the District of Columbia, was conducted. The plans were accessed from the Center for Disease Control from October 1, 2022 to February 28, 2023 [7].

To determine which cancer control plans would be included in the analysis, strict inclusion criteria were established. Specifically, each plan had to have been updated after March 2020 and contain language that specifically referenced the ongoing COVID‐19 pandemic. This approach ensured that the study focused on the most up‐to‐date and relevant policies and strategies developed by state cancer control programs in response to the pandemic.

Following the identification of eligible cancer control plans, a rigorous and systematic thematic analysis was conducted in accordance with best practices and frameworks for qualitative cancer policy research [13, 14, 15, 16]. This involved a detailed examination of the policies and strategies outlined in each plan to identify recurring themes and patterns related to COVID‐19 and its impact on cancer control. The analysis was guided by a comprehensive conceptual framework that incorporated key elements of cancer control policy, including prevention, screening, diagnosis, treatment, and survivorship, as well as the unique challenges and opportunities presented by the COVID‐19 pandemic.

Overall, this approach enabled the identification of important insights and lessons learned related to the intersection of cancer control policy and the COVID‐19 pandemic, which can inform future policy and practice in this critical area of public health. The comprehensive and systematic nature of the analysis ensures that the results of this study are reliable and valid and can be used to guide evidence‐based decision‐making and policy development in the context of the ongoing COVID‐19 pandemic.

## RESULTS

The present study conducted a comprehensive analysis of 51 cancer control plans from all 50 states in the United States, as well as the District of Columbia. Out of these plans, a total of seven were identified as meeting the strict inclusion criteria for the analysis. These states included Illinois (IL), Iowa (IA), Maine (ME), Nevada (NV), North Carolina (NC), Utah (UT), and Vermont (VT) [17, 18, 19, 20, 21, 22, 23]. Two states (ME and VT) only briefly acknowledged the COVID‐19 pandemic and included plans to monitor and evaluate trends in cancer outcomes throughout and beyond the pandemic. The other five states were found to have adapted their cancer control plans in response to the COVID‐19 pandemic along three main themes, including the cancer care continuum, service delivery, and population health and equity (Table 1).

**TABLE 1:** Thematic analysis of state cancer plan response to the COVID‐19 pandemic.

State	Cancer care	Service delivery	Equity
IL	*Screenings*	*Telehealth, telehealth barriers*	*Social determinants of health*
IA	*Screenings, clinical trial participation*	*Workforce shortages, public insurance, public health investment*	*Social determinants of health*
ME	*Plan for future monitoring and evaluation only*		
NV	*Prevention*	*Telehealth*	*Food security, job loss*
NC	*Treatment*	*Workforce shortages, public insurance, public health investment*	
UT		*Telehealth, telehealth barriers*	*Food security, job loss, racism, gender*
VT	*Plan for future monitoring and evaluation only*		

Abbreviations: IA, Iowa; IL, Illinois; ME, Maine; NC, North Carolina; NV, Nevada; UT, Utah; VT, Vermont.

### Cancer care continuum

Specifically, the findings of the study revealed that two states (IL and IA) had adapted their cancer control plans to prioritize the increase of cancer screenings, whereas IA had prioritized increasing clinical trial participation. The IL plan was informed by a series of qualitative interviews, from which many respondents discussed the impact of COVID‐19 on their cancer screening behaviors and socioeconomic status. Given that respondents acknowledged forgoing cancer screening during the pandemic, the new IL plan prioritized “return to screening” initiatives, especially for typically underserved populations. IA specifically cited the COVID‐19 pandemic as rationale for adapting their plan to improve cancer screening and clinical trial participation. The new IA plan prioritized specific screening initiatives related to public awareness and targeted community outreach, removing system barriers to screening, advocating for policies to increase patient access, and enhancing the availability of genetic risk assessments.

One state (NC) had identified the need to increase treatment, whereas another (NV) focused on prevention activities. Neither state plan offered specific details about how the COVID‐19 pandemic motivated the policy adoption or how the adaptations would be prioritized to improve cancer care.

### Service delivery

Regarding service delivery, three states (IL, NV, and UT) had identified telehealth as a priority for addressing cancer care delivery during the pandemic. However, only two of these states (IL and UT) had identified telehealth equity as a significant barrier that required addressing. Additionally, two states (IA and NC) had prioritized addressing workforce shortages and disruptions caused by the pandemic, whereas both health insurance loss and public health investments were identified as service delivery concerns.

Again, IL plan adaptations related to telehealth service delivery and equitable access were driven by qualitative interviews. Many respondents appeared to be unfamiliar and expressed barriers to utilizing telehealth for their cancer care or as caregivers of family members receiving cancer care. Specific plan prioritized included expanding telehealth access by leveraging existing patient navigation systems and improving provider trust and training. A key priority within the plan is to maximize telehealth models to deliver a suite of services (consultations, diagnostic, and treatment) remotely but maintain consistent monitoring by health professionals.

IA's plan explicitly called out the dire effects of the pandemic on workforce shortages and trust in public health; calling for renewed commitments and investment in both aspects of cancer care. One priority created detailed action steps to increase and diversify IA's oncology workforce. Beginning with early education resources to grow the pool of potential healthcare providers to competitive financial packages to support the retention of current physicians and nurses.

Acknowledging the growing importance of telehealth for delivering cancer care across the continuum, the UT plan highlighted how telehealth became an urgent priority during the pandemic. UT's plan also highlighted barriers to telehealth and targeted regions and populations with inequitable access to broadband technology.

### Population health equity

Furthermore, the study found that two states (IL and IA) had acknowledged the critical role of social determinants of health in exacerbating COVID‐19 and cancer inequalities. Additionally, food insecurity (NV, UT) and job loss (NV, UT) were identified as important components of equity within these plans. Notably, UT was found to have explicitly acknowledged the role of racism and gender as related to the disparate impact of COVID‐19 on cancer care.

IL explicitly identified critical strategies for addressing health equity, from increasing awareness by traditional and social media platforms to amplifying leaders in the community addressing health equity in their own communities. The plan also acknowledged the need to address social determinants of health within and outside the healthcare system, with extra emphasis on tracking health disparities data at the community level. Many of these strategies were directly related to the negative effects of the pandemic and barriers to telehealth access.

In other states, there was significant attention to advancing equity within the context of the COVID‐19 pandemic, albeit with less explicit links to strategies and policies. Although not directly tied to the pandemic, IA dedicated an entire chapter of their plan to health equity and acknowledged that the pandemic exacerbated preexisting disparities and social determinants of health among cancer survivors and Iowans at risk of developing cancer. Although NC integrated health equity components throughout their plan, no explicit connection was made between equity and COVID‐19 updates to the plan. Conversely, NV explicitly highlighted the advocacy efforts to improve access to food and mitigate the negative consequences of job loss during the pandemic as a priority and highlight of the new cancer plan. UT's plan highlighted data showing, not only how the pandemic impacted food security, job loss, but also how these negative impacts intersected with existing structural inequities related to racism and gender. The UT plan describes efforts to continue tracking and monitoring data to inform strategies to respond to these negative consequences.

To summarize the state plan adaptations related to the COVID‐19 pandemic, IL described how community feedback led to greater emphasis on leveraging telehealth to expand access to screening and the need to address social determinants of health in and out of the healthcare system. IA, acknowledging the impact of COVID‐19, implemented a set of strategies to improve cancer screenings and clinical trial participation, with service delivery efforts targeting workforce shortages and public health infrastructure. NV's updated plan prioritized prevention, utilizing telehealth services to enhance delivery and address disparities. NC's plan centered on cancer treatment, with service delivery efforts aimed at mitigating workforce shortages and enhancing access to public health resources. UT's plan integrated telehealth services with efforts to address barriers such as food security, job loss, and social inequalities that were created or intensified by the pandemic.

## DISCUSSION

The COVID‐19 pandemic has disrupted cancer care delivery in several ways, including delays in cancer diagnosis and treatment, disruptions to clinical trials, and changes in service delivery models [1, 2, 3, 4, 5]. The pandemic has also highlighted existing disparities in cancer care access and outcomes, which have been exacerbated by the pandemic. Addressing these challenges requires collaboration across various sectors, including healthcare, public health, and policymaking [24]. Collaborative efforts are critical in addressing the challenges posed by the pandemic and ensuring that patients with cancer continue to receive high‐quality care [25].

Before 2020, cancer prevention and control “systems” would be better described as an interconnected but complex web of healthcare providers, insurers, governmental agencies, and nonprofit organizations with heterogeneous incentives to improve health, lower costs, and advance equity [26]. Cancer screening programs were conducted in a variety of settings, including community health centers, private clinics, and specialized screening facilities, each serving different populations based on various levels of healthcare accessibility, as opposed to healthcare need. The type and quality of cancer treatment also depended on factors related to patient preferences, as well as financial and time costs. Despite significant advancements in cancer survivorship, disparities in access to care and outcomes persisted, influenced by factors such as socioeconomic status, race, ethnicity, and geographic location [27]. This recognition fueled a concerted push to address disparities and improve equity across the cancer continuum. With little doubt, the COVID‐19 pandemic further highlighted the need for such targeted interventions and efforts to achieve health equity.

Collaborative efforts to improve cancer care across the care continuum can take several forms. For instance, collaboration among healthcare professionals can facilitate the sharing of best practices and protocols for cancer care delivery during an emergency. Collaboration between healthcare professionals and public health officials can also help to identify and address disparities in cancer care access and outcomes. Moreover, collaboration between policymakers and healthcare professionals can facilitate the development of policies and guidelines that support equitable access to high‐quality cancer care during and beyond the pandemic. Such efforts can help to address the challenges posed by the pandemic and ensure that patients with cancer continue to receive high‐quality care. By working together, healthcare professionals, policymakers, and other stakeholders can create a more resilient and equitable healthcare system that is better prepared to respond to future challenges. State cancer control plans appear to be one venue for facilitating such collaboration.

Many states engaged in collaborative policymaking to improve cancer equity by identifying disparities in social determinants of health, such as food insecurity, housing instability, and access to transportation, employment, and insurance. Recently updated cancer plans also aimed to increase access to preventive services and improve public education about cancer prevention. Only a few of the state plans, however, aimed to identify specific populations that have historically experienced disparities in cancer care or disparate impact from the pandemic [28, 29]. By bringing together various stakeholders and addressing the underlying factors that contribute to cancer disparities, policy collaboration can help to create a more equitable and effective cancer care system.

### Limitations and future directions

This study is not without its limitations. The first limitation relates to the study's generalizability. The generalizability of research on US state cancer control plans may be limited in international contexts due to the substantial involvement of the CDC in funding and supporting plan development, as well as the diverse nature of cancer prevention and control systems worldwide. Moreover, although many plans discuss costs from a patient perspective, there was less attention to how the pandemic impacted costs from a societal perspective or how these adapted strategies may hold up within a cost–benefit analysis. This limitation may seem surprising to an international audience, especially in countries that explicitly require new interventions to be cost‐effective. That attention to cost‐effectiveness is not shared by the US health system. A more notable limitation is the inability to retrospectively analyze prior state plan documentation. This constraint arises due to the unavailability of revision documentation, which, like the plans themselves, is not publicly accessible. Consequently, conducting a retrospective analysis to track the evolution of state plans over time is also limited, which hinders our understanding of how these plans evolve over time. Furthermore, the lack of publicly available documentation related to how these plans are developed underscores the necessity for qualitative research methodologies to elucidate the structures, processes, and outcomes underlying plan development. Qualitative research approaches, such as interviews and case studies, would be indispensable in gaining a comprehensive understanding of the intricacies involved in shaping state cancer plans, including the roles of stakeholders, decision‐making processes, and the contextual factors influencing plan outcomes. Thus, although this current study offers valuable insights into how states adapted their plans due to the COVID‐19 pandemic, the methods and data do not allow us to understand why these changes were made. This inquiry is left for future research.

Many other questions remain unanswered. Specifically, why did some states respond to the COVID‐19 pandemic by updating their plan quicker than others? Are states which are more responsive to external factors, such as the COVID‐19 pandemic, also more responsive to internal factors such as specific subgroup cancer needs? Understanding the process by which these plans are updated could help answer these questions. Research illuminating the mechanisms underlying plan revisions could also provide insights into which plans most effectively address population needs. Through qualitative methods and legal epidemiology analyses, researchers could identify key factors driving responsiveness and effectiveness, whether they be institutional structures, stakeholder engagement, or resource allocation strategies. Finally, examining the relationship between plan development and the ability to meet the state's cancer needs offers valuable insights into the effectiveness of these planning efforts. This research serves as a crucial foundation for refining and optimizing state‐level cancer control strategies, ultimately enhancing their capacity to address the diverse and evolving needs of populations affected by cancer.

## CONCLUSION

The results of this policy analysis provide valuable insights into the ways in which states have adapted their cancer control efforts in response to the COVID‐19 pandemic and may inform future policy decisions at the state and national levels. Some states, such as IL, NV, and UT, expanded their commitment to telehealth delivery. However, each of these three states prioritized unique parts of the cancer control continuum within the broader scope of telehealth (IL‐screening, NV‐prevention, and UT‐social determinants of health). Similarly, even though IA and NC adapted their plan in response to the COVID‐19 pandemic by focusing on commitments to addressing workforce shortages, public insurance, and public health investments, each state prioritized efforts differently (IA‐screenings and clinical trial participation, NC‐treatment). Whether or not these targeted adaptations reflect underlying structural differences, plan development processes differences, or population health outcome differences remains to be seen. Such evidence could illuminate the value of these state plans and how they improve outcomes and address equity. Future policy surveillance can help policymakers evaluate these efforts to prioritize the needs of cancer patients today and ensure that patients receive timely, appropriate care during public health emergencies in the future.

## AUTHOR CONTRIBUTIONS

Conceptualization; investigation; funding acquisition; writing—original draft; writing—review and editing; visualization; validation; methodology; software; formal analysis; project administration; resources; supervision; data curation: Jason Semprini.

## CONFLICT OF INTEREST STATEMENT

The authors declare no conflicts of interest.

## ETHICS STATEMENT

This study used publicly available, secondary data and is not considered Human Subjects Research. There were no animal subjects in this research.

## Data Availability

The data that support the findings of this study are available in CDC Comprehensive Cancer Control Programs at https://www.cdc.gov/cancer/ncccp/ccc_plans.htm. These data were derived from the following resources available in the public domain:—https://www.cdc.gov/cancer/ncccp/ccc_plans.htm, https://www.cdc.gov/cancer/ncccp/ccc_plans.htm
